# Deep learning-based virtual cytokeratin staining of gastric carcinomas to measure tumor–stroma ratio

**DOI:** 10.1038/s41598-021-98857-1

**Published:** 2021-09-28

**Authors:** Yiyu Hong, You Jeong Heo, Binnari Kim, Donghwan Lee, Soomin Ahn, Sang Yun Ha, Insuk Sohn, Kyoung-Mee Kim

**Affiliations:** 1 Department of R&D Center, Arontier Co., Ltd, Seoul, Republic of Korea; 2 The Samsung Advanced Institute for Health Sciences & Technology (SAIHST), Samsung Medical Center, Sungkyunkwan University School of Medicine, Seoul, Republic of Korea; 3 Department of Pathology and Translational Genomics, Samsung Medical Center, Sungkyunkwan University School of Medicine, #81, Irwon-ro, Gangnam-Gu, Seoul, 06351 Korea; 4 Center of Companion Diagnostics, Samsung Medical Center, Seoul, Republic of Korea; 5 Department of Pathology, Ulsan University Hospital, University of Ulsan College of Medicine, Ulsan, Republic of Korea

**Keywords:** Computational biology and bioinformatics, Oncology, Engineering, Mathematics and computing

## Abstract

The tumor–stroma ratio (TSR) determined by pathologists is subject to intra- and inter-observer variability. We aimed to develop a computational quantification method of TSR using deep learning-based virtual cytokeratin staining algorithms. Patients with 373 advanced (stage III [n = 171] and IV [n = 202]) gastric cancers were analyzed for TSR. Moderate agreement was observed, with a kappa value of 0.623, between deep learning metrics (dTSR) and visual measurement by pathologists (vTSR) and the area under the curve of receiver operating characteristic of 0.907. Moreover, dTSR was significantly associated with the overall survival of the patients (*P* = 0.0024). In conclusion, we developed a virtual cytokeratin staining and deep learning-based TSR measurement, which may aid in the diagnosis of TSR in gastric cancer.

## Introduction

With the cancer progression, surrounding tumor microenvironment co-evolves into an activated state and creates a dynamic signaling circuitry promoting cancer initiation and growth, ultimately leading to fatal diseases^1^. Endothelial cells, pericytes, fibroblasts, various inflammatory cells, and the extracellular matrix constitute the stroma surrounding the cancer cells. The tumor–stroma ratio (TSR) represents the proportion of neoplastic cells with respect to tumor-associated stroma. The technique for determining TSR is based on hematoxylin and eosin (H&E) staining of histological sections, allowing the estimation of the amount of stroma present in the primary tumors analyzed using conventional microscopy. The stromal component-predominant TSR-high is an independent adverse prognostic factor in several cancers, including breast cancer (especially triple-negative breast cancer)^2,3^, esophageal squamous cell carcinoma^4^, ovarian cancer^5^, non-small cell lung carcinoma^6^, cervical carcinoma^7,8^, and colorectal carcinoma^9–13^. Despite existing evidence, TSRs are not implemented in routine pathology reports, possibly because of the lack of standard procedure due to varying methodologies used in assessing TSR^14^. Most published studies propose including visual assessments by pathologists; however, such assessments have low inter-observer agreement and reproducibility.

To minimize the problems associated with the visual methods for assessing TSRs, computer-aided deep learning-based methods are being developed to facilitate the automated assessment of TSRs^14,15^. In many studies, computer-aided tumor and stroma quantification, based on the screening of automated tissue segmentation in H&E-stained sections with a combination of hand-crafted features and machine learning, are being used^14–16^. Deep learning algorithms, which are a new branch of machine learning algorithms^17–21^, have recently entered the field of computational pathology, and have shown promise in automating histopathological image analysis^14–16,22–27^. Geessink et al.^14,15^ proposed a deep learning-based TSR scoring, which requires labor-intensive patch-wise or pixel-wise annotations of the tumor, stroma, and normal tissue regions, and this automatic TSR score achieved a moderate agreement of 0.521 kappa compared with two observers’ consensus scores. Xu et al*.*^27^ recently introduced a conditional CycleGAN^18^ to transform H&E images into immunohistochemical (IHC) images by transferring the styles. However, the CycleGAN mechanism uses unpaired H&E and IHC images during the training stage; the generated IHC images may provide less precise information than the actual IHC images. Furthermore, Bulten et al*.*^22^ and Tellez et al*.*^23^ used IHC images as reference standards to extract annotations for specific cells using a typical classification model.

In the present study, we aimed to distinguish tumors from the stroma using simplified binary annotated masks without laborious annotation. For this purpose, we developed a deep learning model with pixel-level-paired H&E and IHC images using a single network.

## Materials and methods

### Patients

This study was approved by the Institutional Review Board of Samsung Medical Center (approval number: 2020-04-225) and included a consecutively selected cohort of 373 patients with stage III and IV gastric adenocarcinoma. All patients underwent curative surgery in 2014 and 2015 at the Samsung Medical Center (Republic of Korea), and written informed consent was obtained from all participants. All experiments were performed in accordance with the relevant guidelines and regulations. No patient received neoadjuvant chemotherapy or died within 30 days from the date of surgery. Prior to surgery, no distant metastases or other cancers in patients were diagnosed. Follow-up clinical data were obtained from medical records. Clinicopathological findings, including age, sex, Lauren type, pathology, depth of tumor invasion (pT), and lymph node metastasis (pN) stages, are listed in Table 1.

**Table 1:** Clinicopathologic characteristics of patients with gastric adenocarcinoma.

	Training set (n = 13)	Test set for TSR assessment (n = 358)
	(Mean ± SD)	(Mean ± SD)
Age	53 (± 11.71)	64 (± 12.88)
**Sex**		
Male	10 (76.92%)	230 (64.25%)
Female	3 (23.08%)	128 (35.75%)
**Lauren type**		
Intestinal	7 (53.85%)	76 (21.23%)
Diffuse	6 (46.15%)	225 (71.23%)
Mixed	0 (0.00%)	0 (0.00%)
Indeterminate	0 (0.00%)	27 (7.54%)
**Pathology**		
Hepatoid adenocarcinoma	0 (0.00%)	1 (0.28%)
Mucinous adenocarcinoma	0 (0.00%)	27 (7.54%)
Signet-ring cell carcinoma	1 (7.69%)	36 (10.06%)
Tubular adenocarcinoma, well/moderately differentiated	7 (53.85%)	76 (21.23%)
Tubular adenocarcinoma, poorly differentiated	5 (38.46%)	218 (60.89%)
**AJCC stage**		
Stage III	7 (53.85%)	163 (45.53%)
Stage IV	6 (46.15%)	195 (54.47%)

*SD* standard deviation.

### Preparation of multiplexed H&E- and IHC-stained sections and scanning

Slides were prepared for a representative section of each of the 373 formalin-fixed and paraffin-embedded (FFPE) tissue samples; FFPE tissue section (4-µm-thick) were prepared and stained with H&E using an automatic stainer for routine diagnostic purposes. Representative slides containing deeply invasive tumor parts were selected to visually assess the TSR and tumor pT stage.

For multiplexed H&E- and IHC-stained sections, IHC staining for cytokeratin (CK) (Novocastra™ Liquid Mouse Monoclonal Antibody with 1:500 dilution) was performed. Intermediate filaments found in epithelial cells of all types and markers for carcinoma cells were first analyzed using BOND-MAX Autoimmunostainer (Leica Biosystems, Melbourne, Australia). The IHC-stained slides were scanned at × 200 total magnification (tissue-level pixel size, 0.32 μm/pixel) with Aperio Digital Pathology Slide Scanner (Aperio Technologies, Inc., Vista, CA). After scanning, the same slides were stained with H&E after removing aminoethylcarbazole (AEC) by washing with distilled water, and then, the slides were incubated with 70%, 80%, and 95% ethanol for 2 min each, followed by incubation with 0.15 M KMnO_4_ for 1 min as previously described^20^. A total of 15 pairs of multiplexed H&E and CK whole-slide images (WSIs) of gastric carcinoma were prepared for this study.

### Dataset preparation

As shown in Table 1, we prepared WSI datasets consisting of one training dataset and two test datasets for evaluation. Each pair of H&E and CK WSI or only H&E WSI was obtained from each patient. The training dataset, which contained 13 pairs of H&E and CK WSIs, was used to train a deep learning model about structured context between H&E and CK images.

One test dataset, which contained two pairs of H&E and CK WSIs, was mainly used for the visual comparison of virtually generated CK image against the real CK-stained image. The other test dataset that contained the 358 H&E WSIs was used to evaluate the performance of our TSR-scoring method. The test datasets were used as hold-out sets and were not used for model training or optimization.

### Visual measurement of TSR (vTSR) by the pathologists

The TSR score for each specimen was visually assessed by experienced pathologists (B. K. and K.-M. K.). Areas with the highest amount of stroma were selected, and the amount of stromal tissue was estimated per 10% increment using a 10× objective lens. The TSR was categorized into vTSR-high (> 50% stroma) and vTSR-low (≤ 50% stroma) groups based on the recommended guidelines^9^. In cases where the stroma region was larger than the tumor region in hotspots, the WSI was scored as vTSR-high, and in cases where the tumor region was larger than the stroma region in hotspots, the WSI was scored as vTSR-low.

### Development of a deep learning pipeline

**Figure 1:**
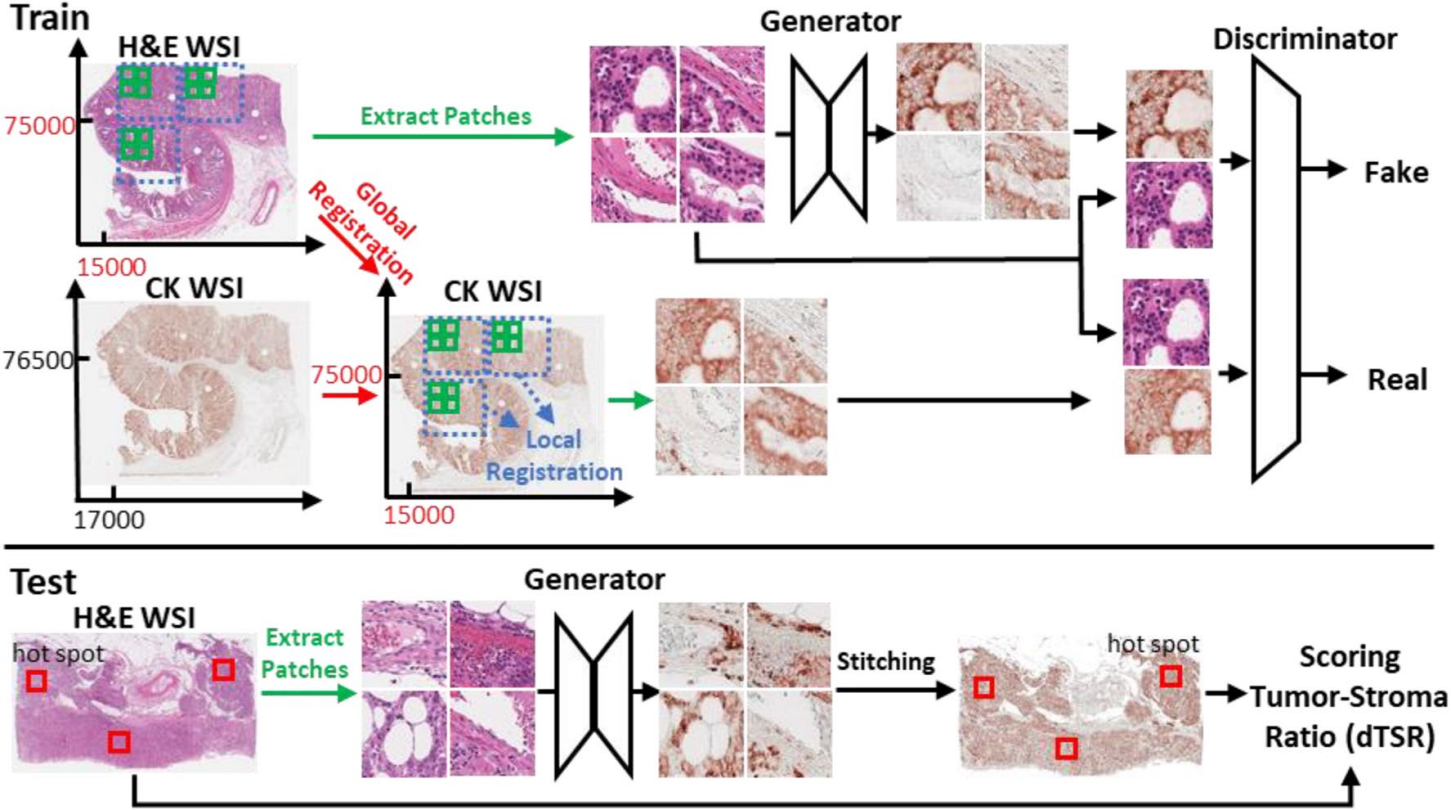
The pipeline of our proposed method.

The pipeline for our method is illustrated in Fig. 1. At the training stage, we registered CK and H&E images to ensure high-pixel-level similarity. The process was performed in a global and local manner. After registration, each corresponding local region was tiled into pairs of small patch images to train cGAN. The use of deep adversarial training allowed the model to learn structural information and details between H&E images and their corresponding CK images. During model testing, the H&E patch images were inputted into the training generator to generate the CK image. The generated CK images were stitched into a part of or an entire WSI for further scoring of the dTSR using corresponding H&E images.

### Registration between H&E and CK images

**Figure 2:**
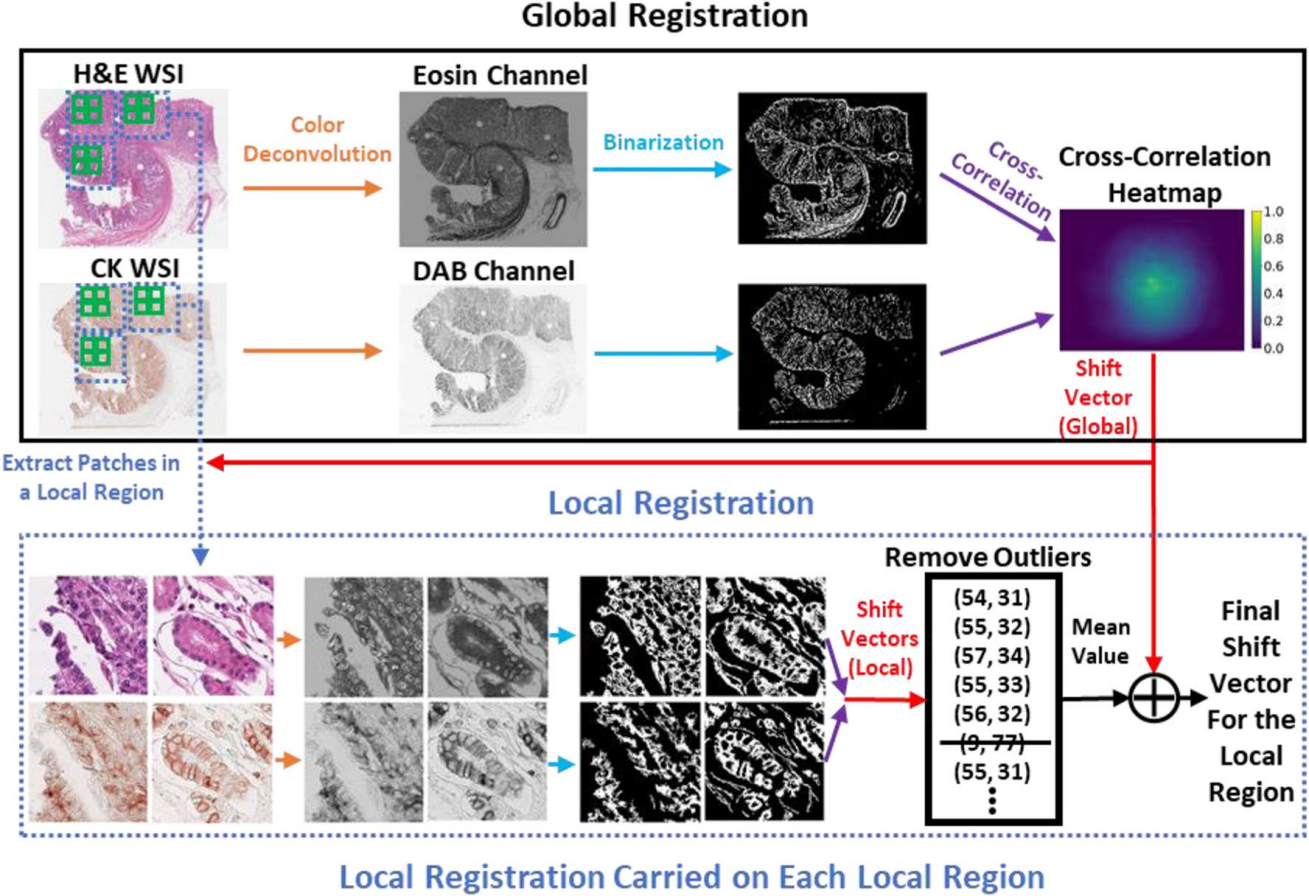
The pipeline of our image registration process.

Figure 2 shows the pipeline of our registration method, which is a two-step (global and local) method. A global shift vector was calculated to coarsely align the pair of WSIs. A local shift was then calculated for each local region to enable precise registration. A detailed description of the registration method is as follows. First, the H&E and CK WSIs were downsampled 32 times. Next, color deconvolution^28^ was performed on both sets of images to transform their RGB color space into a hematoxylin-eosin-diaminobenzidine (HED) color space. CK antibody is mainly stained in the cytoplasm; therefore, eosin color channels were extracted from H&E images, and diaminobenzidine (DAB) color channels were extracted from CK images. Thereafter, we binarized the two channel images using locally adaptive thresholding to establish the binary threshold for small regions of the images. Each threshold value was calculated as the weighted mean of the local neighborhood subtracted by an offset value. The local neighborhood block size was set at 151, and the offset value was set at 10. After binarization, small objects with area under 200 were removed from the binary images. The global shift vector was calculated by determining the location of the maximum value of the 2D cross-correlation heatmap of the two binary images. Cross-correlation can be calculated by employing the fast Fourier transform algorithm. To achieve precise mapping between the two types of images, local registration must be performed in succession. Global shifting was applied onto the images of the local regions with the same location as shown in WSIs and with 10,240 × 10,240-pixel resolution, and then, the images were extracted in a tiling manner. From each local region, we further extracted patch images with 1024 × 1024-pixel resolution also in a tiling manner, except for those with almost all-white backgrounds. The local shift vector of each pair of patch images was calculated using the same process used for calculating the global shift vector. The mean of the local shift vectors, which allowed the removal of outliers with local region plus the global shift vector, were established as the final shift vector for the local region.

### CK image generation using H&E images using Pix2Pix cGAN

After registration, a one-to-one correspondence between H&E and CK patch images was achieved. To generate CK images from H&E images, a model should learn the structured context between the two images. In other words, a model should learn the semantic information between CK and H&E images during the transformation process. If a model could preserve high semantic correctness, it would establish virtually generated CK images into functional data as the generated images would function like the actual stained image. Through a thorough investigation, we considered the Pix2Pix deep generative adversarial architecture^18^ as the best approach for this task. Such a model is sufficiently flexible in detecting subtle differences in a range of higher-order statistics between generated and real CK images. The Pix2Pix model is trained using deep adversarial learning that can automatically learn a proper loss function instead of through manually engineered loss functions.

Included in the Pix2Pix architecture is a generator network $G$ and a discriminator network $D$. From a previous study^18^, the generator was a U-net^19^-shaped network with skip connections, and the discriminator was based on a PatchGAN that operates by classifying individual patches in an image as real or fake. By importing an H&E image $x$ into the generator, a CK image $G(x)$ was generated. Furthermore, by defining real CK images as $\mathrm{y}$, the discriminator $D$ learned to classify between real $\{x, y\}$ pairs and fake $\{x, G(x)\}$ pairs. The generator was trained to develop CK images that could not be distinguished from real CK images by an adversarial trained discriminator. Both the generator and discriminator observed the input H&E images, and the discriminator used pairs from both real and fake images.

To provide noise such that the network could produce a high stochastic output, dropout was applied onto the generator in the original Pix2Pix architecture. However, in our case, the network required more predictable and deterministic outputs; thus, we removed the dropout from our generator network.

The objective of our conditional GAN was formulated as follows:

1 $${\mathcal{L}}_{cGAN}\left(G, D\right)={\mathbb{E}}_{x,y}\left[\mathrm{log}D(x,y)\right]+{\mathbb{E}}_{x}\left[\mathrm{log}(1-D(x,G(x)))\right],$$

where $G$ minimized this objective against an adversarial $D$ that attempted to maximize it. The generator $G$ attempted to reduce the loss from the discriminator and additionally attempted to move the fake distribution close to the real distribution using the $L1$ loss. Besides the $L1$ loss in the RGB color space, we added $L1$ loss into the HED color space between the real CK image and fake CK image.

2 $${{\mathcal{L}}_{L1}\left(G\right)}^{RGB}={\mathbb{E}}_{x,y}\left[{\Vert {y}^{RGB}-{G(x)}^{RGB}\Vert }_{1}\right],$$

3 $${{\mathcal{L}}_{L1}\left(G\right)}^{HED}={\mathbb{E}}_{x,y}\left[{\Vert {y}^{HED}-{G(x)}^{HED}\Vert }_{1}\right].$$

Our final loss function is

4 $${G}^{*}=\mathrm{arg} \; \underset{G}{\mathrm{min}} \; \underset{D}{\mathrm{max}}{\mathcal{L}}_{cGan}\left(G, D\right)+ {\lambda }_{1}{{\mathcal{L}}_{L1}\left(G\right)}^{RGB}+ {\lambda }_{2}{{\mathcal{L}}_{L1}\left(G\right)}^{HED}.$$

The network was implemented in PyTorch (https://pytorch.org/). During training, ${\lambda }_{1},$ ${\lambda }_{2}$, and the batch size were set to 10, 0.9, and 1, respectively. The Adam optimizer with a fixed learning rate of 0.0002 was used to minimize the loss function. The model was trained for 200 epochs. At the test stage, the discriminator was removed, and only the generator was used to develop the CK images from the H&E images. The code developed and used for this study can be found at https://github.com/YiyuHong/ck_virtual_staining_paper.

The images used for training and inference were processed as follows: H&E and CK WSI were all tiled into 256 × 256-pixel resolution patch images at × 200 total magnification (tissue-level pixel size 0.32 μm/pixel) using OpenSlide library (https://openslide.org) after image registration processing. The pairs of patch images in the white background that are H&E patch images where > 95% of the pixel values were above 220 in the gray scale color space were removed^25,26^. Many CK patch images with little dyed areas (no epithelial cells) were not useful for the model to learn structural contexts between H&E and CK images. These types of pairs of images were downsampled and accounted for approximately 10% of the total training images. A CK patch image was considered as having little dyed area if < 5% of pixels were left on the DAB channel that was thresholded at 80.

### Measurement of TSR by the deep learning metrics (dTSR)

**Figure 3:**
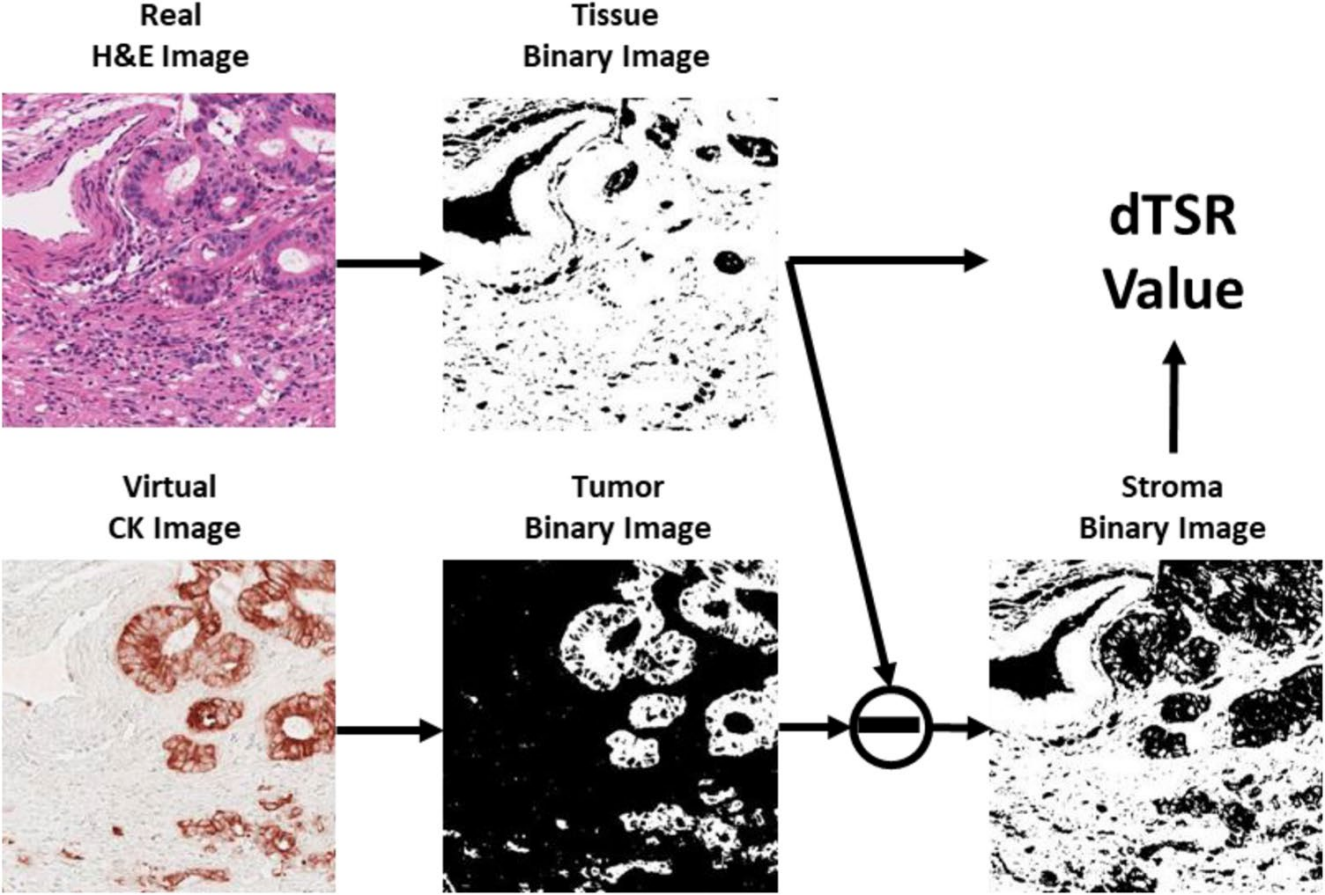
The dTSR scoring process. The white region on the binary image indicates the corresponding detected region of tissue, tumor, and stroma.

The overall process to calculate the dTSR score using virtually generated CK images is shown in Fig. 3. First, real H&E image and virtual CK image pairs were binarized. The H&E image was binarized by thresholding at 200 after transformation into grayscale. The CK image was binarized by thresholding with 80 on the DAB channel that was obtained by color deconvolution^28^. In the figure, the white region of the binarized H&E image and the binarized virtual CK image were regarded as the tissue region and tumor region, respectively. We considered the removed tumor region from the tissue region as the stroma region. The dTSR in this study was calculated using the following equation:

5 $$dTSR= \frac{Stroma \,area}{Tumor \,area+Stroma\, area}\times 100\%.$$

## Results

### Visual assessment of registered H&E and CK images

**Figure 4:**
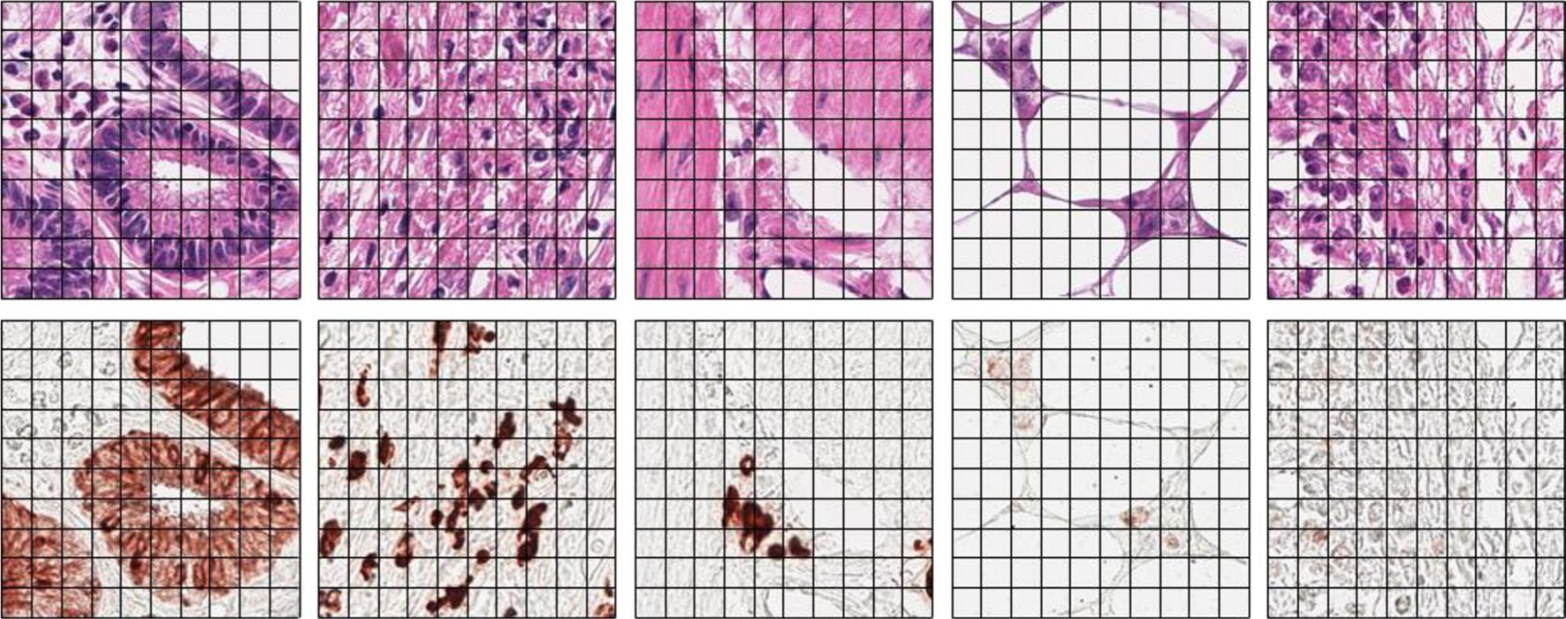
Pairs of hematoxylin and eosin (H&E) and cytokeratin (CK) staining patch images after registration.

As both H&E and CK WSI were available from the re-staining of the same tissue sections, we found almost no complex nonlinear deformations; however, we did find a simple vertical and horizontal shift between them. These shifts have also been previously reported^22,23^. Figure 4 shows the representative pairs of H&E and CK patch images after registration. All images were from different WSIs. By following the black grid in the figure, only minor deformations between the H&E images and their corresponding CK patch images were observed.

### Visual comparison of virtual and real CK staining

**Figure 5:**
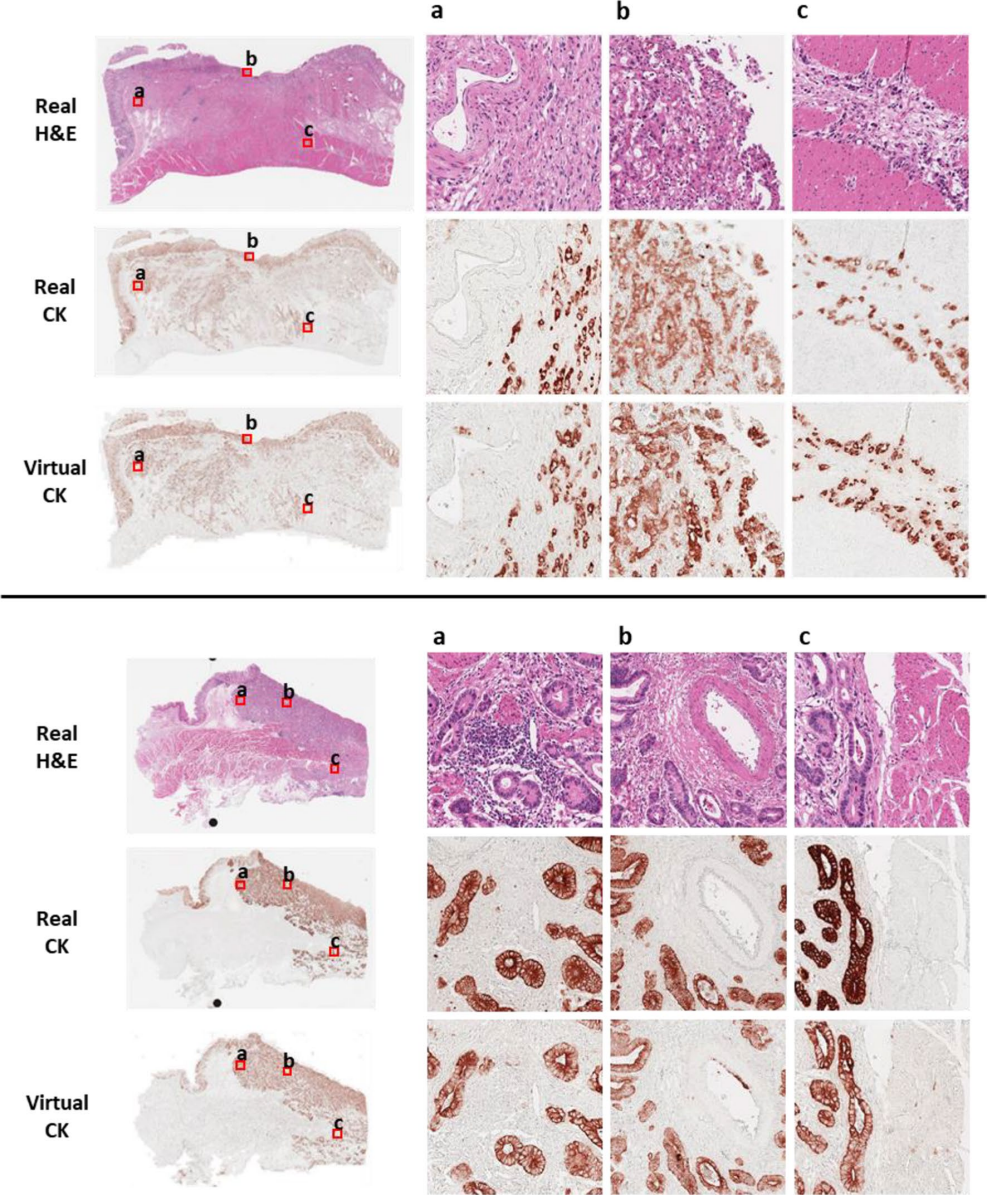
Visual comparison of real hematoxylin and eosin (H&E)- and real cytokeratin (CK)-stained images against virtual CK-stained images at the WSI and patch level.

Figure 5 shows the real H&E images, real CK images, and virtual CK images at the WSI and patch levels for visual comparison. Real H&E and CK images were obtained from the test dataset for visual comparison. The real CK images were obtained after the registration of the real H&E images, and the virtual CK images were obtained by transforming the corresponding real H&E images using our method. At the WSI level, the overall staining pattern between the real CK WSI and virtual CK WSI was similar: scattered single tumor cells and cancer glands were stained in the same cells, although the staining intensity was different among areas within the tumor. Interestingly, vessels mimicking tumor glands were not visualized by virtual CK staining. The high sensitivity and specificity of virtual CK staining observed using the visual comparison of images indicated that our trained model had learned the difference in semantic context between H&E and CK images.

**Figure 6:**
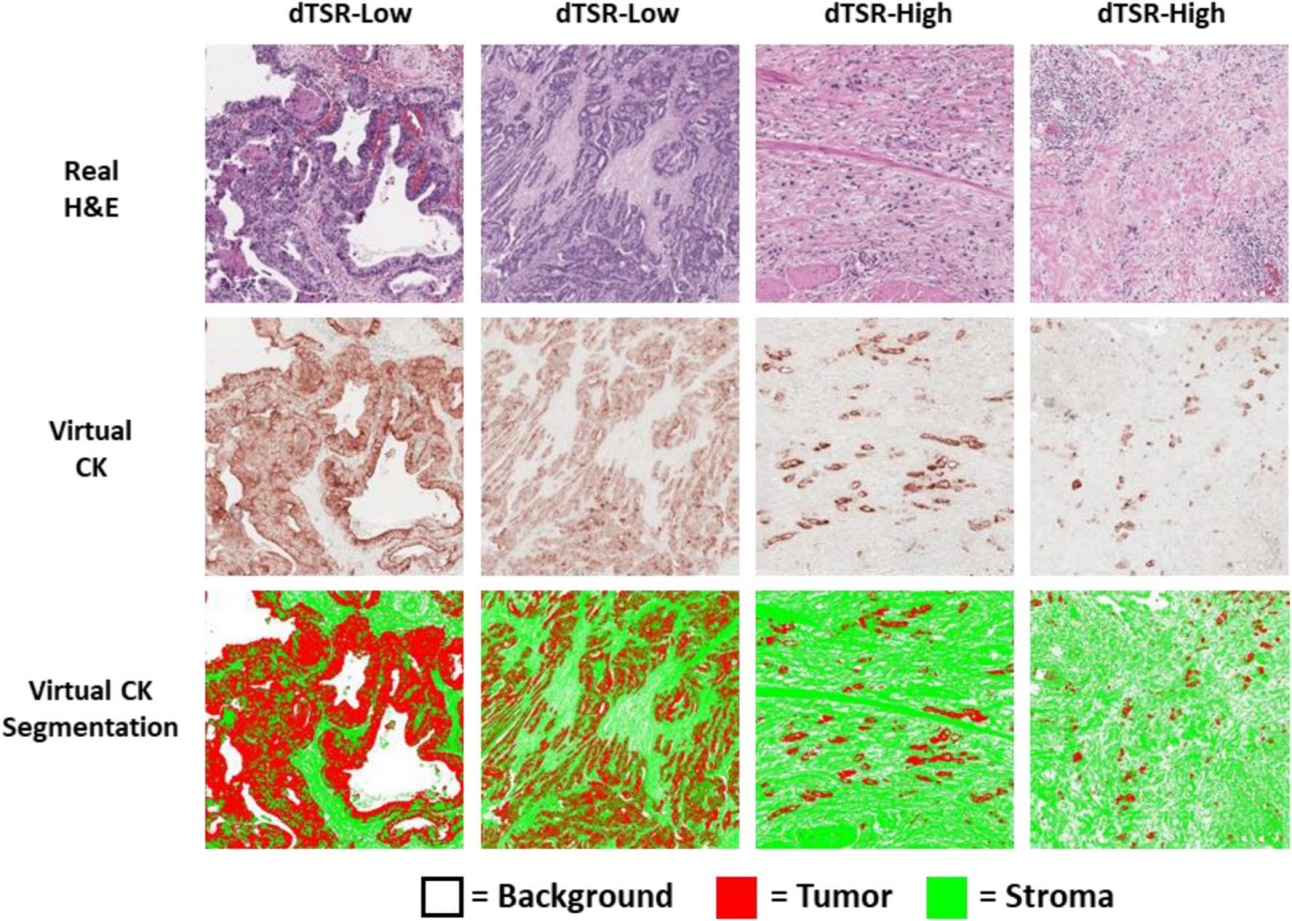
Tumor and stroma segmentation results on virtual CK images that were transformed from hematoxylin and eosin (H&E)-stained images on the hotspots of tumor–stroma ratio (TSR) assessment. The two columns on the left are images of dTSR-low, whereas the two columns on the right are images of dTSR-high.

To ensure accurate TSR assessment of the tumor cell-rich areas using our method, segmentation performance for tumor and stroma on the virtual CK images must be checked. Figure 6 shows the dTSR assessed using H&E images from the test dataset for TSR scoring. They were transformed into virtually generated CK images using our method and segmented as shown in Fig. 3. The segmented images showed that the tumor region occupied most of the area in the segmented dTSR-low images compared with the segmented dTSR-high images. Although other types of cells and structures in the stroma region could be present because the stroma region was obtained by removing the tumor region from the tissue region, they were negligible with respect to the TSR-scoring hotspots.

### Agreement of TSR between deep learning and pathologists and its effect on prognosis

In this study, wherein deep learning-based measurement was applied, 358 advanced gastric cancers were divided into 121 (33.80%) dTSR-low and 237 (66.20%) dTSR-high tumors. According to the visual assessments by the pathologists, the 358 gastric cancers comprised 128 (35.75%) vTSR-low and 230 (64.25%) vTSR-high tumors. Agreement between dTSR and vTSR was measured using the test dataset for TSR scoring, where the dataset included H&E WSIs of 358 gastric carcinomas (Table 2). Each WSI was labeled as either vTSR-low or vTSR-high by pathologists, and the hotspots marked with tumor-rich and deeply infiltrating and invasive cancer areas were annotated on each WSI. For WSI with several hotspots, our method for calculating dTSR scores for each of the hotspots was used, and the average dTSR score was assigned to the WSI.

**Table 2:** Correlations between dTSR and vTSR and corresponding clinicopathologic characteristics.

	Test set for TSR assessment (n = 358)		*P*-value
	dTSR-low	dTSR-high	
	(n = 121)	(n = 237)	
**Age**			0.0006
< 60	28 (23.14%)	100 (42.19%)	
≥ 60	93 (76.86%)	137 (57.81%)	
**Sex**			0.0121
Male	89 (73.55%)	141 (59.49%)	
Female	32 (26.45%)	96 (40.51%)	
**Lauren type**			< 0.0001
Intestinal	47 (38.84%)	29 (12.24%)	
Diffuse	61 (50.41%)	194 (81.85%)	
Indeterminate	13 (10.75%)	29 (5.91%)	
**Pathology**			< 0.0001
Hepatoid adenocarcinoma	1 (0.83%)	0 (0.00%)	
Mucinous adenocarcinoma	13 (10.74%)	14 (5.91%)	
Signet-ring cell carcinoma	8 (6.61%)	28 (11.81%)	
Tubular adenocarcinoma, well/moderately differentiated	46 (38.02%)	30 (12.66%)	
Tubular adenocarcinoma, poorly differentiated	53 (43.80%)	165 (69.62%)	
**AJCC stage**			0.0195
Stage III	66 (54.55%)	97 (40.93%)	
Stage IV	55 (45.45%)	140 (59.07%)	
**vTSR**			< 0.0001
Kappa = 0.623			
vTSR-low	98 (80.99%)	30 (12.66%)	
vTSR-high	23 (19.01%)	207 (87.34%)	

**Figure 7:**
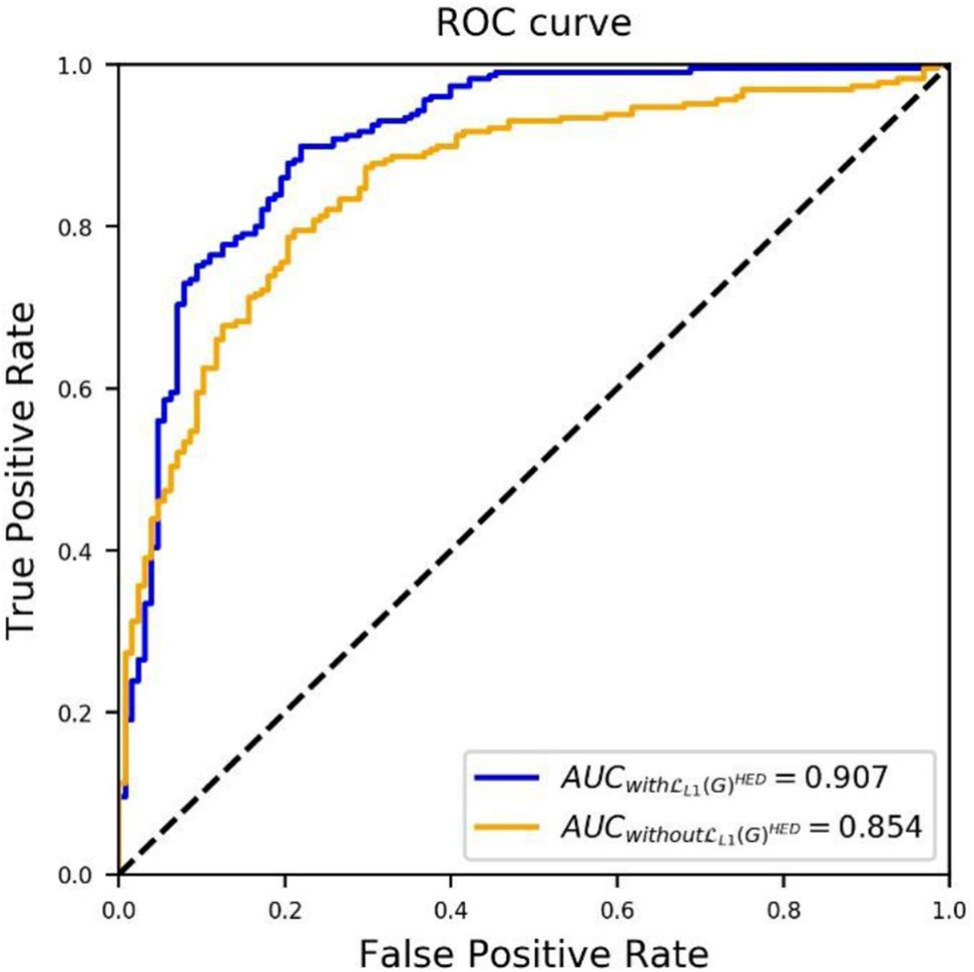
Receiver operating characteristic (ROC) curve of dTSR-scoring performance of the proposed method without and with ${{\mathcal{L}}_{L1}\left(G\right)}^{HED}$.

Low and high TSRs scored by the pathologists were treated as true labels and set as 0 and 1, respectively, to calculate the ROC and AUC curves of the proposed method, as shown in Fig. 7. Adding the ${{\mathcal{L}}_{L1}\left(G\right)}^{HED}$ loss improved the performance of the proposed method compared to using only ${{\mathcal{L}}_{L1}\left(G\right)}^{RGB}$ loss, thereby resulting in an AUC of 0.907 (improved by 0.053) for the test dataset. An AUC of > 0.90 indicates that our method has a high performance in automatically assessing TSR-low and TSR-high WSI in hotspots.

Cohen’s kappa was also calculated to measure the TSR-scoring agreement between the dTSR and vTSR. After assessing several cut-offs, such as 50%, 55%, 65%, 70%, and 75%, for dichotomizing dTSR into dTSR-high and dTSR-low, the 65% cut-off was identified to provide the best kappa value. The details are presented in Table 2. A substantial agreement, with a kappa of 0.623, was found.

**Figure 8:**
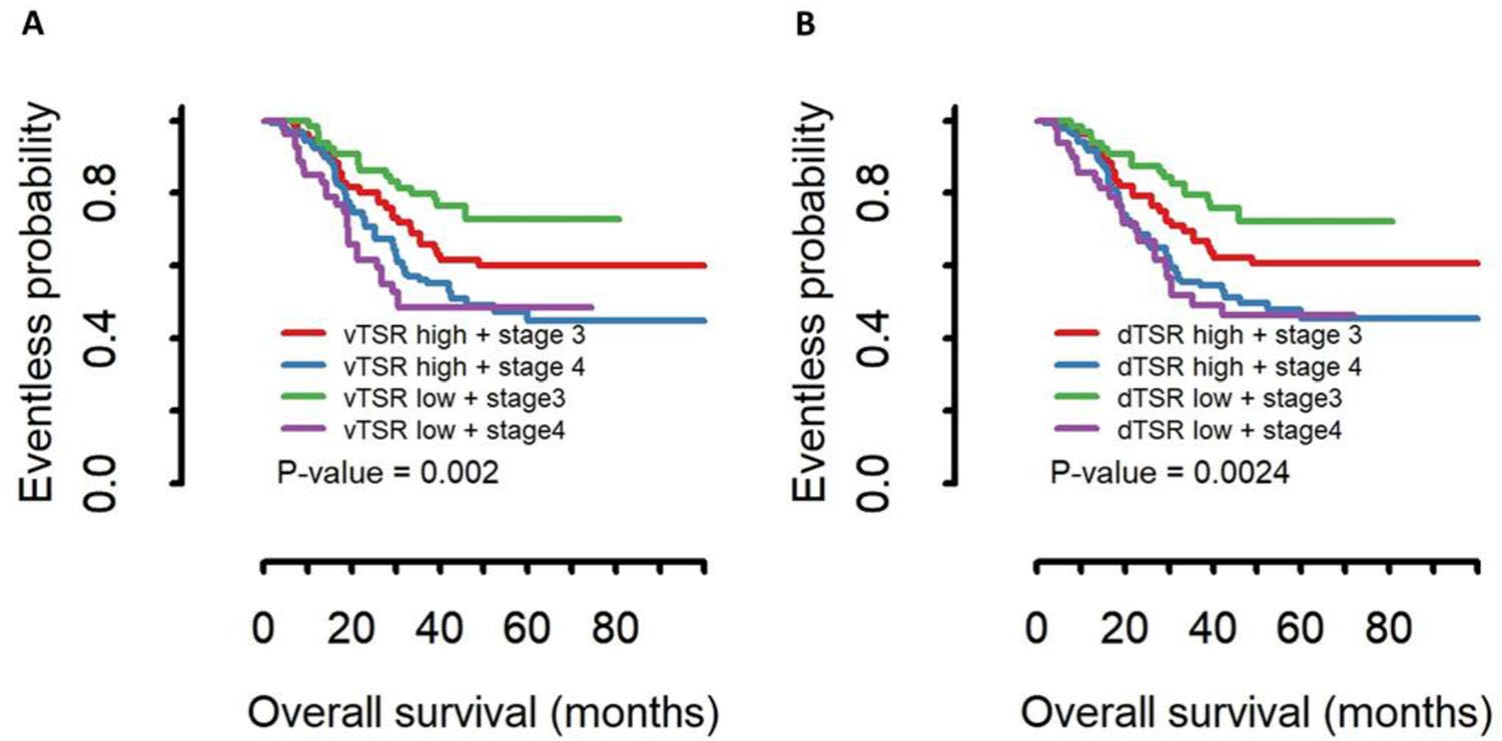
Kaplan–Meier curve of the overall survival of patients stratified by tumor–stroma ratio (TSR) measured by pathologists (vTSR) (**A**) and TSR using the deep learning metrics (dTSR) (**B**) for gastric cancers.

Parameters of the correlation between dTSR with clinicopathological characteristics are described in Table 2. Tumors classified as dTSR-high tumors were significantly associated with a diffuse histologic subtype based on higher disease stage (*P* = 0.0195), pathology (*P* < 0.0001), Lauren type (*P* < 0.0001), and vTSR (*P* < 0.0001). In predicting patient overall survival, dTSR-high tumors were significantly associated with worse overall survival in stage III and IV cancer patients; the statistical values for the predictive power of dTSR (*P* = 0.0024) and vTSR (*P* = 0.002) were similar (Fig. 8). We constructed a Cox proportional hazards model and found that dTSR-high was associated with worse prognosis of patients (hazard ratio 1.268, 95% 1.125–11.23, *P* = 0.0307).

## Discussion

In this study, we developed a deep learning-based TSR measurement tool, aided by virtual CK staining using combined image registration techniques and a conditional GAN-based generation model. Using deep learning-based dTSR measurement, 358 advanced gastric cancers were classified into 121 dTSR-low and 237 dTSR-high tumors; the dTSR-high tumors were significantly associated with advanced disease stage and worse overall survival in patients with stage III and IV cancer. Moreover, the agreements for determining TSR using dTSR and vTSR yielded a moderate agreement with an AUC value of 0.907. Moreover, in patients with stage III gastric cancer, dTSR was more significantly associated with the overall survival of patients than vTSR.

Tumor stroma is an integral part of cancer initiation, growth, and progression. Moreover, stromal elements of tumors hold prognostic and response-predictive information, and abundant targeting opportunities within the tumor microenvironment continue to be identified^1^. Consistent with previous studies on various tumor types^2–12^, we first identified that TSR-high tumors were a poor prognostic factor for advanced gastric cancer. Although we failed to determine any significance in stage IV gastric cancer patients, determining a prognostic factor for stage IV gastric cancers was difficult owing to poor prognosis wherein 5-year survival rates reportedly range from 15 to 18%^29^. In our previous molecular classification of gastric cancer^30^, mesenchymal-like molecular subtype was closely associated with poor prognosis and diffuse type according to Lauren classification. In the present study, compared with TSR-low subtypes, we demonstrated that the TSR-high subtype was significantly associated with diffuse histologic subtypes and worse prognosis.

Lou et al.^31^ provided evidence that TSR upon the initial diagnosis of ovarian carcinoma was associated with the eventual emergence of its resistance to platinum-based chemotherapy. They also indicated that TSR is straightforward and not cost-prohibitive as it utilizes routine histopathologic slide evaluations and is a parameter that can be included in pathology reports as part of medical records. TSR can be further validated by examining tissues and correlating it with the outcomes of large-scale co-operative group trials^31^. Variations in methodology and a lack of a standard procedure in assessing TSR^14^ have posed challenges in establishing routine pathology reports. To address such challenges, standardized assessment methods without inter-observer variability are important. The recent introduction of digital pathology in routine tissue diagnostics provides opportunities for automated TSR analysis^14,15^. Herein, we used deep learning-based dTSR measurements and found that it correlated with vTSR and prognostic significance.

In the present study, image registration and de-staining and re-staining processes for the same tissue sections for H&E and CK staining of WSIs guaranteed pixel-level registration with minimal shifting changes. Image registration with high performance enabled the production of large amounts of training image data that subsequently improved the performance of the deep learning model. Although some registered patch images with small shifting errors were observed, their effect on the model performance was insignificant owing to their small quantity. Alternatively, similar to normal processes in pathologic research and diagnosis, tissue sections can be stained with H&E and CK to produce training data. However, as they are not from an identical tissue section, significant morphological differences make the pixel-level registration almost impossible; deep learning models hardly learn structured context from these kinds of paired images.

The approach of using a manual pixel-wise annotation and IHC staining as a reference standard can be assessed based on two aspects: annotation precision and labor intensity. For example, in the present study, it was possible to annotate the epithelial tumor cells that were tightly attached in clusters in the H&E image; however, it was almost impossible to precisely annotate all individually scattered epithelial tumor cells as it required enormous labor. Furthermore, subjectivity affected the classification of the cell types, with ambiguous morphology and introduced intra- and inter-observer variability, resulting in reduced precision. By contrast, the CK IHC annotation was more objective and precise in marking epithelial tumor cells, although slight artifacts during scanning and non-specific staining could reduce precision. The main labor requirement came from the destain–restain process for producing IHC WSIs, which was laborious and costly. Based on the findings of recently published studies^22,23,27^ that utilized IHC WSI with deep learning, we trained a generative model by introducing CK IHC WSI for TSR scoring instead of using manual annotations^14,15^. The TSR-scoring ability was verified by correlating the results of our method with those obtained by experienced pathologists from this large dataset (AUC of 0.907 and kappa of 0.623 on 358 gastric carcinoma H&E WSI). Moreover, direct comparisons of real CK images and virtual CK images detected almost the same cells with high precision in both intestinal and diffuse type gastric carcinomas. Validation of the proposed technology on a larger and independent dataset is essential for the technology to be incorporated into routine pathologic diagnostics. The objectivity of a deep learning-based TSR measurement method, which allows accurate and reproducible quantification, has the potential to pave the way for the implementation of TSR in clinical practice^14^.

Nevertheless, there are two main limitations of the present study: (1) the selection of hotspots for TSR measurement is not automatic; instead, selection was subjectively performed by a pathologist. Therefore, pathologists using our approach would result in different TSR scores as they may select different hotspots on the same WSI. To solve this problem, an objective and automatic hotspot detection module is required. (2) As our deep learning model was trained using data from a single institute, it may not be usable on datasets from other hospitals. Tissue samples that are processed, stained, and prepared differently may contain different color tones and morphological details that would decrease the performance of this deep learning model. To overcome these limitations, we plan to develop a fully automatic TSR measurement approach that includes automatic hotspot detection to reduce inter-pathologist variation. Moreover, we intend to implement prospective, multi-institutional consortium to train a more robust deep learning model.

Until recently, TSR was evaluated by pathologists; however, the use of H&E slides and TSR score prediction techniques using deep learning has just begun, although it has limited accurate prediction power. Our study presents a novel technique to evaluate TSR using a deep learning model with virtual CK images generated using cGAN by inputting H&E slide images.

## Data Availability

The datasets generated and/or analyzed in the current study are available from the corresponding author upon reasonable request.

## Abbreviations

H&E: Hematoxylin and eosin staining
WSI: Whole-slide image
CK: Cytokeratin staining
DAB: Diaminobenzidine
TSR: Tumor–stroma ratio
vTSR: Visual tumor–stroma ratio
dTSR: Digital tumor–stroma ratio
ROC: Receiver operating characteristic
AUC: Area under the ROC curve

## Code availability

The code used in this manuscript can be found on Github at https://github.com/YiyuHong/ck_virtual_staining_paper.
